# Improvement of Docetaxel Efficacy through Simultaneous Blockade of Transcription Factors NF-κB and STAT-3 Using Pentoxifylline and Stattic in Prostate Cancer Cells

**DOI:** 10.3390/cimb46090605

**Published:** 2024-09-14

**Authors:** José Roberto Cruz-Lozano, Georgina Hernández-Flores, Pablo Cesar Ortiz-Lazareno, Luis Arturo Palafox-Mariscal, Katia Carolina Vázquez-Ibarra, Karen Lilith González-Martínez, María Martha Villaseñor-García, Alejandro Bravo-Cuellar

**Affiliations:** 1 Doctoral Program in Biomedical Sciences, University Center of Health Sciences, University of Guadalajara, Guadalajara 44340, Mexico; jroberto.cruz@alumnos.udg.mx; 2 Immunology Division, Western Biomedical Research Center, Mexican Institute of Social Security, Guadalajara 44340, Mexico; georgina.hernadezf@imss.gob.mx (G.H.-F.); pablo.ortiz@imss.gob.mx (P.C.O.-L.); luisarturopalafox@gmail.com (L.A.P.-M.); kc.vazquezi@gmail.com (K.C.V.-I.); lilith.gonzalez.m@gmail.com (K.L.G.-M.); 3 Department of Pharmacobiology, University Center of Exact Sciences and Engineering, University of Guadalajara, Guadalajara 44430, Mexico; 4 Department of Health Sciences, University Center of Los Altos, University of Guadalajara, Guadalajara 47620, Mexico

**Keywords:** prostate cancer, tumor progression, drug resistance, docetaxel, pentoxifylline, Stattic

## Abstract

Prostate cancer (PCa) is a common and deadly disease in men. It is often diagnosed at advanced stages, at which point patients are treated mainly with docetaxel (DTX), which is effective but limited by resistance and side effects. Overactivation of the transcription factors NF-κB and STAT-3 plays a critical role in the development, progression, and chemoresistance of PCa. In this regard, the blockade of NF-κB with pentoxifylline (PTX) or STAT-3 with Stattic (STT) is known to increase the sensitivity of tumor cells to chemotherapy in both in vitro and in vivo models. We investigated whether simultaneous blockade with PTX and STT increases the efficacy of the DTX treatment in inducing apoptosis in metastatic castration-resistant PCa DU-145 cells. Our results showed that the combination of PTX + STT led to higher levels of apoptosis, regardless of whether or not DTX was present in the treatment. Determining caspases and ΔΨm indicates that the intrinsic caspase pathway of apoptosis is principally favored. In addition, this combination inhibited proliferation and colony formation and arrested the cell cycle in the G1 phase. These results indicate that the combination of the PTX + STAT-3 inhibitor could potentiate DTX effectively, opening the possibility of effective treatments in PCa.

## 1. Introduction

Prostate cancer (PCa) is the second most frequent cancer and the fifth cause of death in men worldwide, with an estimated 1.47 million new cases and 396,792 deaths registered in 2022 [1]. PCa does not exhibit noticeable symptoms in its early stages but gradually progresses over several years. As a result, a high percentage of patients are diagnosed with advanced-stage PCa, where they are commonly treated with androgen deprivation therapy (ADT) [2]. However, this disease shows heterogeneous behavior, and this form of therapy is frequently insufficient. Patients stop benefiting from it as PCa becomes castration-resistant (CRPC), and mortality rates increase simultaneously. In this setting, chemotherapy is necessary to prevent excessive proliferation of PCa cells [3,4]. In the majority of cases, first-line chemotherapy for patients involves the use of taxanes, particularly docetaxel (DTX) (Table 1), which has shown some benefit when used initially in conjunction with some forms of hormonal therapy, such as abiraterone, enzalutamide, or more recent antiandrogens [5,6,7]. However, its severe adverse effects often lead to poor tolerance in most patients. It is important to mention that if DTX becomes ineffective or ceases to work, cabazitaxel offers survival benefits for those with progressive disease, although it does not improve pain palliation [8,9]. Consequently, research is ongoing to explore new alternatives that can enhance current treatments, improve outcomes, and minimize the side effects of antitumor therapy.

Major players in PCa progression are the transcription factors STAT-3 and NF-κB. Both tend to demonstrate the overexpression commonly associated with the more aggressive forms of this disease, exhibiting higher Gleason scores (≥7), and their constitutive activation has been reported in metastatic niches of patients showing null response to treatment [4,10,11,12]. In addition, it has been demonstrated that both transcription factors turned on in the same cancer cells and associated stroma, where they co-regulate numerous oncogenes responsible for increasing proliferation and promoting survival, thus avoiding cell apoptosis, as well as those related to enhancing their invasive, angiogenic, and immunosuppressive capacities [13,14]. Interestingly, studies suggest that both factors are involved in CRPC development and DTX resistance acquisition [9,15]. Given the critical role of both factors in exacerbating this disease, inhibitors targeting these pathways have become of great interest in combating highly metastatic CRPC.

In this regard, Stattic (STT) (Table 1) is a non-peptide molecule that potently and selectively inhibits the phosphorylation of the tyrosine 705 (Y705) of the transcription factor STAT-3, thus preventing its translocation to the nucleus [16]. Hence, STAT-3 inhibition by STT has been reported to increase apoptosis in several in vitro studies on nasopharyngeal, esophageal, ovarian, and prostate cancer cell lines [17,18,19,20], as well as in vivo xenograft models of colorectal cancer, diffuse large B-cell lymphoma, and prostate cancer [21,22,23]. Importantly, it has been demonstrated that STT favorably modifies the tumor microenvironment, prompting a shift in macrophage phenotype from M2 to M1, in this manner increasing macrophage toxicity against tumor cells [24].

For its part, pentoxifylline (PTX) (Table 1) is a semi-synthetic methylxanthine-derived drug approved by the U.S. Federal Drug Administration (FDA) for treating peripheral vascular disease. Most interestingly, extensive clinical and experimental evidence supports its potential for repurposing as an antineoplastic agent because PTX inhibits the nuclear translocation of NF-κB by preventing phosphorylation of its natural inhibitor IκBα at serines 32 and 36, stabilizing the IκBα-NF-κB complex in the cytoplasm and thus avoiding the NF-κB active survival genes and inflammatory cytokines, including TNF-α [25]. In vitro and in vivo studies have shown that PTX significantly increases apoptosis, either alone or in sensitizing tumor cell lines to the toxic action of chemotherapy agents such as Adriamycin, cisplatin, and carboplatin on various types of cancer [26,27,28]; moreover, when PTX was combined with Adriamycin in lymphoma-bearing mice, a 100% survival rate was achieved with only one half the dose of Adriamycin considered effective in murine models [26]. In the clinic, it has been reported that PTX induced remission and increased apoptosis in pediatric patients with acute leukemia during the steroid window phase [29]. Additionally, in previous work, we reported that the exclusive use of PTX against metastatic PCa PC3 cells induces more apoptosis than DTX itself, and the best results were obtained when both drugs were combined [30].

Thus, following the concept of chemotherapy with a rational molecular basis, in the present work, we investigated whether the simultaneous inhibition of NF-kB and STAT-3 is better than inhibiting a single factor to increase DTX-induced apoptosis in PCa metastatic human CRPC DU-145 cells.

## 2. Materials and Methods

### 2.1. Cell Line and Culture Conditions

The human prostate cancer cell line DU-145 was obtained from the American Type Culture Collection (HTB-81™; ATCC, Manassas, VA, USA). These cells were cultured in Roswell Park Memorial Institute 1640 (RPMI) culture medium supplemented with 10% heat-inactivated fetal bovine serum (FBS), 1% L-glutamine solution, and 100 IU/mL of the penicillin–streptomycin–neomycin (PSN) antibiotic mixture (all Gibco™; Thermo Fisher Scientific, Waltham, MA, USA). This latter culture medium will hereinafter be referred to as RPMI-S. Cells were maintained in a humidified atmosphere containing 5% CO_2_ at 37 °C. Upon reaching 80% confluency, cells were washed with phosphate buffered saline (PBS), pH 7.4 (Gibco™; Thermo Fisher Scientific, Waltham, MA, USA) and harvested using Accutase (Sigma-Aldrich, St. Louis, MO, USA). Cell viability was determined by Trypan Blue 0.4% (Sigma-Aldrich, St. Louis, MO, USA), exclusion (viability > 95%) prior to all experiments, and then the cells were seeded at different densities to perform the assays. Cells were used within 10 passages to maintain similarity to the original tumor cells.

### 2.2. Drugs and Reagents

PTX (Sigma-Aldrich, St. Louis, MO, USA) was dissolved in RPMI-1640 culture medium at a concentration of 250 mM and stored at 4 °C for fewer than 8 days. STT was reconstituted in DMSO to create 50 mM stock solutions (all Merck KGaA, Darmstadt, Germany) and stored in aliquots at −80 °C for fewer than 8 days until use. DTX (Pisa Pharmaceutical, Guadalajara, México) was dissolved in a sterile saline 0.9% solution and maintained at −80 °C. The chemotherapeutic agents were dissolved immediately prior to each experiment.

### 2.3. In Vitro Treatments

**Figure 1:**
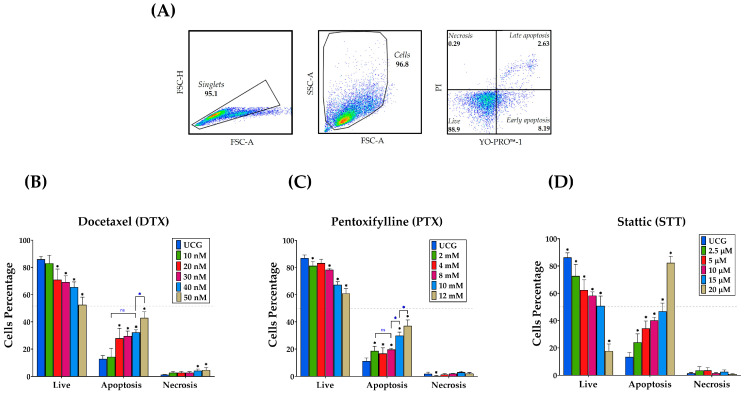
Assessment of the apoptotic effect of PTX, STT, and DTX at different concentrations in DU-145 cells. Apoptosis (late + early) was determined by flow cytometry using YO-PRO-1 and PI dual staining after 48 h of the different treatments: (**A**) flow cytometry analysis strategy, (**B**) DTX, (**C**) PTX, and (**D**) STT. Results are represented as the mean ± the SD of three independent experiments, each carried out in triplicate. Statistical analysis: Mann–Whitney *U* test, * *p* < 0.05 vs. UCG (untreated control group) and * *p* < 0.05 in the comparison between groups; ns = not significant.

DU-145 cells were cultured overnight at 37 °C and 5% CO_2_ before treatments to allow cells to attach to the plates. After that, cells were treated with DTX (50 nM), PTX (10 mM), and STT (15 μM) or their simultaneous combinations. In all experiments, PTX was added 1 h before treatment with DTX or STT; this treatment schedule yielded better results [26]. An untreated control group (UCG) was employed as a negative control for this study. The doses selected were determined through dose–response curves for the apoptotic effect after 48 h of treatment (Figure 1).

### 2.4. Apoptosis

Under similar experimental conditions, apoptosis was assessed by flow cytometry using dual staining with YO-PRO-1 and propidium iodide (PI) (Invitrogen-Thermo-Fisher, Waltham, MA, USA). For this purpose, DU-145 cells (2 × 10^5^) were seeded in 12-well plates and treated according to the previously mentioned description for 48 h. The cells were then harvested with Accutase and washed twice with PBS. Subsequently, staining with YO-PRO-1 and PI was performed for 30 min at room temperature and protected from light. A minimum of 10,000 events were analyzed for each sample by the Cytoflex cytometer (Beckham-Coulter^®^, Brea, CA, USA), and data were processed using FlowJo V10 software (Tree Star, Inc., Ashland, OR, USA). Data are expressed as the mean ± the standard deviation (SD) of the positive percentage of early plus late apoptosis cells, calculated as follows: live cells = (−) for both YO-PRO-1 and PI, early apoptosis cells = YO-PRO-1 (+) and PI (−), late apoptosis cells = (+) for both YO-PRO-1 and PI, and necrotic cells = YO-PRO-1 (−) and PI (+).

### 2.5. Proliferation Assay

Proliferation was determined using the BrdU Cell Proliferation ELISA Kit (Abcam, Cambridge, UK). DU-145 cells (1 × 10^4^) were seeded in 96-well plates and exposed to the different treatments for 48 h; 24 h prior to the period of incubation, BrdU was added. After aspirating the cell culture, a fixing solution was added, and the cells were incubated. A wash with wash buffer was performed, followed by the addition of an anti-BrdU monoclonal detector antibody, and the cells were incubated for 1 h at room temperature. Another wash was conducted, and peroxidase goat anti-mouse IgG conjugate was added, with subsequent incubation and a third wash. Tetramethylbenzidine (TMB) peroxidase substrate was added, and after incubation in the dark, stop solution was introduced. Optical density was measured at 450/540 nm using a microplate reader (Synergy™ HT; BioTek^®^ Instruments, Inc., Winooski, VT, USA). Results are reported as the mean ± the SD of the proliferation percentages obtained in each group.

### 2.6. Colony Number Formation Assay

To evaluate the effect on colony formation after short-term treatments, the following was performed: 3 × 10^5^ cells were seeded in 6-well plates, and once attached, were exposed to 4 h treatments. Later, cells were harvested using Accutase and washed with PBS (to discard all dead cells). The cell button was then resuspended in 1 mL of RPMI-S culture medium, and then live cells were counted using the CytoSMART™ cell counter (Axion Biosystems^®^, Atlanta, GA, USA). Finally, 300 cells were seeded in a new 6-well plate and incubated under standard cell culture conditions. Growth was monitored for 15 days, and at the end of this period, the formed colonies were fixed with 3.7% formaldehyde and stained with sulforhodamine B to be counted using Image J software version number 1.54i. (NIH; Bethesda, MD, USA) and expressed as a number of colonies, in comparison with the UCG group.

### 2.7. Assessment of Pan-Caspase Activity

In that apoptosis is related to caspase activity, pan-caspase activity was determined by flow cytometry using the Generic Caspases Activity Assay-Fluorometric Green Kit (Abcam, Cambridge, UK) according to the manufacturer’s instructions. DU-145 cells (2 × 10^5^ cells) were cultured and treated as in the apoptosis experiment. After harvesting and washing, the cells were resuspended in RPMI-S culture medium in cytometry tubes. The fluorescent caspase inhibitor TF2-VAD-FMK was added, and the mixture was incubated for 2 h at 37 °C. Following two washes with PBS, cells were resuspended in assay buffer for reading on the Cytoflex cytometer (Beckman-Coulter^®^, Brea, CA, USA) considering 10,000 cell-region events per experiment. Data are expressed as the mean ± the SD of the mean fluorescence intensity of the positive expression of pan-caspase activity and analyzed using FlowJo V10 software (Tree Star, Inc., Ashland, OR, USA).

### 2.8. Evaluation of Caspase-3, Caspase-8, and Caspase-9 Activity

To evaluate the involvement of caspase-3, -8, and -9, DU-145 cells (2 × 10^5^) were cultured in 12-well plates and treated for 48 h. Activation of each of these proteins was determined by flow cytometry employing Caspase-3 (active), Caspase-8 (active), and Caspase-9 (active) FITC Staining Kits (Abcam, Cambridge, UK). Cells were harvested, washed, and incubated in RPMI-S culture medium with appropriate fluorescent inhibitor conjugates (DEVD-FMK for caspase-3, IETD-FMK for caspase-8, and LEHD-FMK for caspase-9) for 1 h at 37 °C. Finally, cells were washed two times with PBS and resuspended with wash buffer for acquisition on the Cytoflex cytometer (Beckham-Coulter^®^) considering 10,000 cell-region events. The data obtained were analyzed using FlowJo V10 software (Tree Star, Inc., Ashland, OR, USA), and the results were represented as the mean ± the SD of the mean fluorescence intensity of the positive expression of the activity of each caspase in the different groups.

### 2.9. Determination of Mitochondrial Membrane Potential (Δψm)

Due to the central role that the mitochondria might play in the execution of apoptosis, we assessed the loss of Δψm using JC-10, a cationic and lipophilic reagent (Abcam, Cambridge, UK). DU-145 cells (1 × 10^4^) were seeded in a 96-well optical black wall transparent bottom plate (Nunc-Thermo Fisher, Rochester, NY, USA) and treated for 48 h with the different drugs. Afterward, the cells were stained with the JC-10 reagent according to the manufacturer’s instructions. In apoptotic cells that have lost their Δψm, JC-10 fails to accumulate as aggregates inside the mitochondria that emit orange fluorescence (590 nm), instead existing as monomers, which emit green fluorescence (530 nm). Cells were analyzed using the microplate reader (Synergy™ HT; BioTek^®^ Instruments, Inc., Winooski, VT, USA). Results are presented as the mean ± the SD of the fluorescence values normalized to the UCG group for each respective group.

### 2.10. Cell Cycle Analysis

For cell cycle analysis, DU-145 cells were synchronized through gradual serum depletion. Thus, 1 × 10^6^ cells were seeded in 25 cm^2^ culture flasks in the RPMI-1640 culture medium supplemented with 5% FBS and incubated for 12 h. Later, cells were maintained with an RPMI-1640 culture medium supplemented with 1% FBS for another 12 h. After this period, the cells remained in a serum-free RPMI-1640 culture medium for 18 h. At the end of the depletion, the cells were stimulated by adding RPMI-S to continue their cell cycle process, and from this moment on, the different treatments were applied for 48 h according to the scheme. Finally, the cells were harvested and washed for labeling with the BD Cycletest™ Plus DNA Kit (BD Biosciences^®^, San Jose, CA, USA) according to the instructions of the supplier. At room temperature, the cells were incubated with trypsin buffer for 10 min. Then, trypsin and RNase inhibitor buffer were added, and another incubation time was allowed under the same conditions. Last, the PI stain was added, and the cells were incubated for 10 min in the dark at 2–8 °C. At least 30,000 events were analyzed on the Cytoflex cytometer (Beckham-Coulter^®^). Data were analyzed using FlowJo V10 software (Tree Star, Inc., Ashland, OR, USA), and the results were expressed as the percentage of cells in the G1, S, and G2 phases.

### 2.11. Determination of Pro-/Antiapoptotic Gene Expression by Real-Time qRT-PCR

To investigate the changes in the expression of pro- and anti-apoptotic-related genes, DU-145 cells (3 × 10^6^) were seeded in 100 mm Petri dishes with 10 mL of RPMI-S culture medium under standard cell culture conditions. After 16 h of incubation, the medium was replaced with fresh medium containing the treatments under study according to each group, and incubated for 4 h. Total RNA extraction was then performed as indicated in the specifications of the RNeasy^®^ Plus Mini Kit (Qiagen, Hilden, Germany). Five μg of total RNA were taken to perform the cDNA synthesis utilizing the commercial Superscript™III First-Strand Synthesis SuperMix Kit (Invitrogen, Waltham, MA, USA). Real-time or quantitative polymerase chain reaction (qPCR) was performed using the LightCycler^®^ 1.5 Kit and the LightCycler^®^-FastStart DNA MasterPLUS SYBR Green I Kit (Roche, Mannheim, Germany). All samples were processed in duplicate. In order to normalize the expression data, *RPS18* and *RPLP0* were utilized as reference genes. The results were analyzed with LightCycler Version 4.1 software (Roche Applied Science). The relative expression levels were determined using the 2−ΔΔCp algorithm and converted into Log2FC. Oligonucleotides were designed employing the nucleotide database of the National Center for Biotechnology Information GenBank (http://www.ncbi.nlm.nih.gov, accessed on 17 March 2024) using Oligo version 6 software and are shown in Table 2.

### 2.12. Phosphorylation State of p65 and STAT-3 Transcription Factors

The phosphorylation status of p65 and STAT-3 in DU-145 cells was detected using flow cytometry. For this purpose, 1 × 10^6^ cells were treated with the different treatments for 1 h. The cells were then harvested with Accutase, washed with PBS, resuspended in fixation buffer (BioLegend, San Diego, CA, USA), and incubated for 20 min at 4 °C. Once fixed and washed, the cells were permeabilized with Intracellular Staining Perm Wash Buffer (BioLegend, San Diego, CA, USA) for 5 min at room temperature. Afterward, primary rabbit IgG Ab against anti-phospho-NF-κB-p65 (Ser536, dilution 1:800; Cell Signaling Technology, Inc., Boston, MA, USA) or anti-phospho-STAT-3 (Tyr705, dilution 1:500; Abcam, Cambridge, UK) was added and incubated for 30 min. Subsequently, the cells were washed, and secondary Ab goat-anti-rabbit IgG-FITC (1 μg per 1 × 10^6^ cells; Santa Cruz Biotechnology, Inc., Santa Cruz, CA, USA) was added and incubated again for a further 30 min. Finally, the cells were washed and resuspended in PBS for the acquisition on the Cytoflex cytometer (Beckman-Coulter^®^) of 10,000 cell-region events. We used cells stained exclusively with the secondary Ab to eliminate the background. Data analysis was performed using FlowJo V10 software (Tree Star, Inc., Ashland, OR, USA), and the results are expressed as percentage expressions and geometric mean fluorescence intensity (MFI).

### 2.13. Statistical Analysis

All experimental procedures were performed in triplicate and repeated at least three times. Results are expressed as the mean ± the standard deviation (SD) of the values obtained. Statistical analyses were performed on GraphPad version 8.0.2 software (San Diego, CA, USA) using the Mann–Whitney *U* test to calculate group differences. Statistically significant differences were considered when the *p* value was <0.05.

## 3. Results

### 3.1. Effect of PTX, STT, and DTX Drugs on the Induction of Apoptosis in Metastatic Prostate Cancer DU-145 Cells

Initially, we determined the concentrations of the DTX, PTX, and STT to be used through a dose–apoptotic effect curve after 48 h of treatment. For this purpose, we chose the following concentrations: 10, 20, 30, 40, and 50 nM for DTX; 2, 4, 8, 10, and 12 mM for PTX; and 2.5, 5, 10, 15, and 20 μM for STT. Thus, we selected the concentrations of 50 nM for DTX, 10 mM for PTX, and 15 μM in the case of STT, these being the concentrations that came nearest to inducing 50% of cells in apoptosis, with 42.9%, 29.7%, and 46.7%, respectively (Figure 1B–D). These doses are in agreement with those that have been employed to date.

### 3.2. Combinations of Pentoxifylline + Stattic + Docetaxel Led to the Enhanced Induction of Apoptosis in Metastatic Prostate Cancer DU-145 Cells

**Figure 2:**
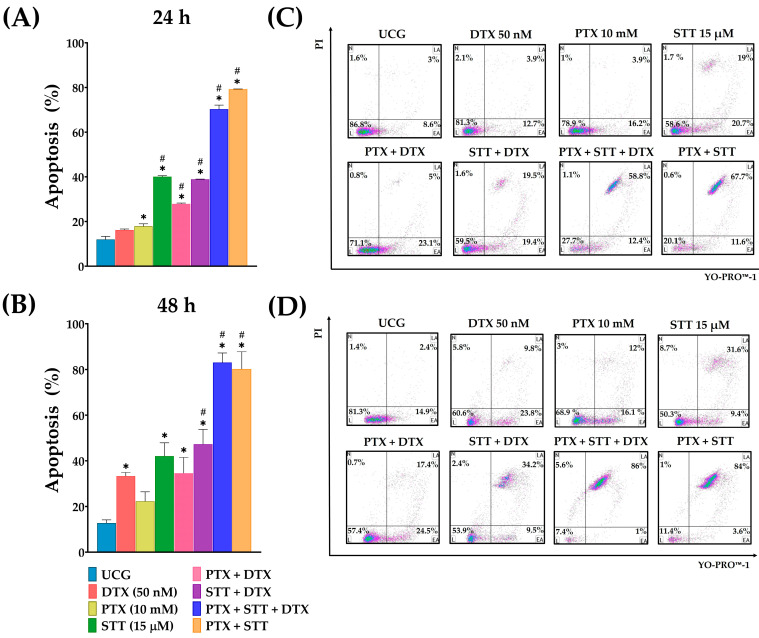
Effect of PTX, STT, DTX, or a combination on the induction of apoptosis in DU-145 cells. UCG = untreated control group; DTX = docetaxel; PTX = pentoxifylline; STT = Stattic. Percentage of DU-145 cells in apoptosis (late + early) determined by flow cytometry after dual staining with YO-PRO-1 and PI following exposure with 50 nM of DTX, 10 mM of PTX, and 15 μM of STT, as well as combinations, for 24 (**A**) or 48 h (**B**) of incubation. The results are represented as the mean ± the SD of three independent experiments, each carried out in triplicate. Statistical analysis: Mann–Whitney *U* test, * *p* < 0.05 vs. UCG; # *p* < 0.05 vs. DTX 50 nM; (**C**,**D**) representative dot plots from one of the three independent experiments of each of the treatments at both times.

After establishing the standard conditions for the experiments, in order to confirm our hypothesis, we investigated the effect of the different drug combinations to induce apoptosis. At 24 h of incubation, the use of isolated drugs showed a slight increase in apoptosis for the PTX and DTX groups. On the other hand, higher levels of apoptosis were achieved by the STT-treated group (40%), confirming previous results. Remarkably, when combining the drugs, higher levels of apoptosis were obtained compared to those of the UCG or DTX group, with the most prominent groups being those treated with PTX + STT + DTX and PTX+ STT, with 70.3% and 79.2% of apoptosis, respectively (Figure 2A,C). When the experiment was performed after 48 h of incubation, all groups demonstrated an increase compared to UCG (Figure 2B,D); furthermore, at this time, the STT group and the combination of STT + DTX exerted a significant effect, with similar apoptosis percentages between them compared to the group treated with DTX alone. Finally, the PTX + STT + DTX and PTX + STT groups continued to reveal the highest percentages of apoptosis, with values near those of the 24 h experiment.

### 3.3. The Combined Treatment of Pentoxifylline + Stattic + Docetaxel Inhibits DU-145 Cellular Proliferation

**Figure 3:**
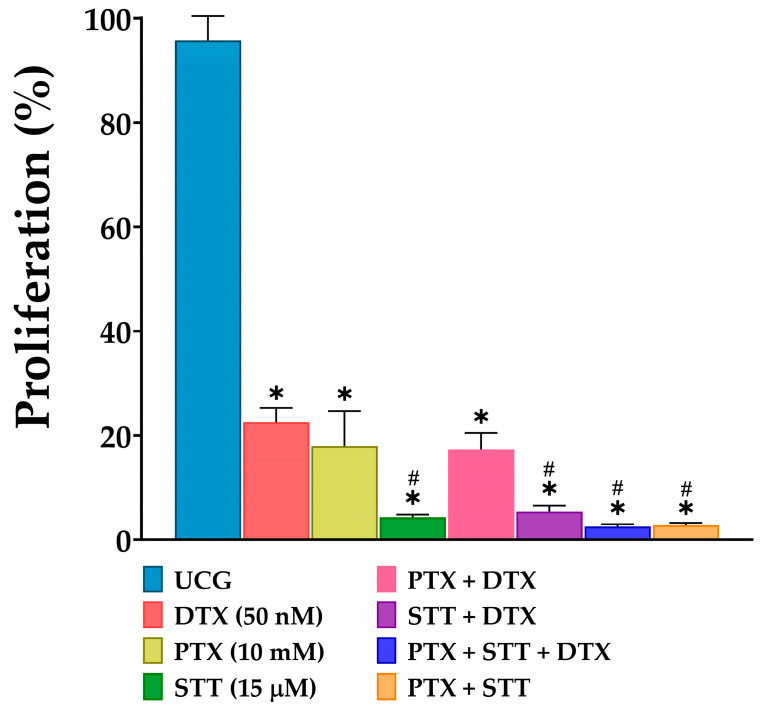
Effect of PTX, STT, DTX, or a combination on the proliferation of DU-145. UCG = untreated control group; DTX = docetaxel; PTX = pentoxifylline; STT = Stattic. The proliferation percentage of cells was measured by spectrophotometry after treatment with 50 nM of DTX, 10 mM of PTX, and 15 μM of STT or combinations for 48 h. Data are represented as the mean ± the SD of three independent experiments, each carried out in triplicate. Statistical analysis: Mann–Whitney *U* test, * *p* < 0.05 vs. UCG and # *p* < 0.05 vs. DTX 50 nM.

One crucial characteristic of cancer cells is their uncontrolled proliferation. Therefore, we evaluated the effect of different treatments on DU-145 cells after 48 h. In this regard, all treated groups were significant compared to the UCG (*p* < 0.05). Interestingly, we observed that the groups with STT exhibited the greatest decrease in proliferation, with close percentages. This effect was evident in the PTX + STT combination and in the experimental group, with 98% proliferation inhibition; the remaining treated groups demonstrated an inhibition of cell proliferation of close to 80% (Figure 3). These results correlate with those of previous apoptosis assays: With STT, whether DTX is present or not, greater cytotoxicity is achieved.

### 3.4. Pentoxifylline + Stattic per se and Combined with Docetaxel Blocked the Clonogenic Capacity of DU-145 Cells

**Figure 4:**
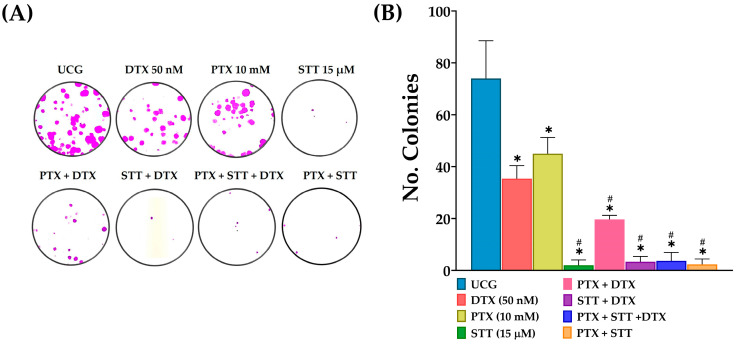
Effect of PTX, STT, and DTX or a combination on the clonogenic capacity of DU-145. UCG = untreated control group; DTX = docetaxel; PTX = pentoxifylline; STT = Stattic. Cells were treated with 50 nM DTX, 10 mM PTX, and 15 μM STT, as well as a combination, for 4 h. Dead cells were discriminated, and live cells were seeded. After 15 days of culture, colony-forming ability was assessed. (**A**) Representative images of DU-145 cells stained with sulforhodamine B (**B**) and their quantification from three independent experiments are shown. Data are represented as the mean ± the SD. Statistical analysis: Mann–Whitney *U* test, * *p* < 0.05 vs. UCG and # *p* < 0.05 vs. DTX 50 nM.

Clonogenic capacity was determined to assess the capacity of DU-145 cells to form colonies after exposure to the treatments. The results revealed a significant effect after exposure to the treatments in a short time period of 4 h. The ability to form clones or new colonies decreased after treatment exposure. This effect was more significant (*p* < 0.05) in the groups treated with STT individually and combined with PTX or DTX, compared with UCG, which maintained a high clonogenic capacity. On the other hand, a moderate decrease was observed when cells were treated with the combination of PTX + DTX, and weaker effects were observed with PTX and DTX alone. However, they continued to show differences concerning UCG (Figure 4).

### 3.5. Caspase Activity from DU-145 Cells after In Vitro Treatment with Docetaxel, Pentoxifylline, and Stattic or Combinations

**Figure 5:**
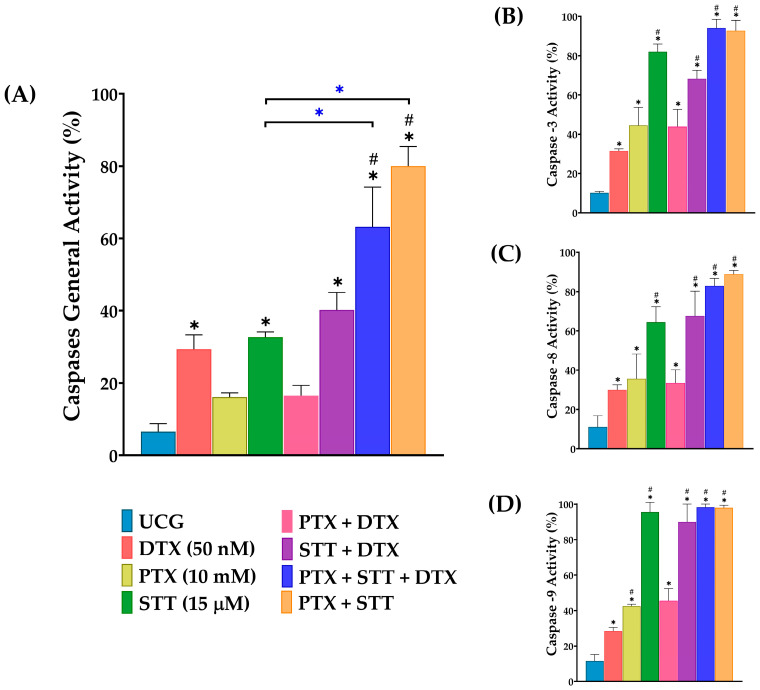
Effect of PTX, STT, DTX, or a combination on caspase activity in DU-145 cells. UCG = untreated control group; DTX = docetaxel; PTX = pentoxifylline; STT = Stattic; (**A**) percentage of pan-caspase activity, (**B**) caspase-3, (**C**) caspase-8, and (**D**) caspase-9 activities measured by flow cytometry after exposure with 50 nM of DTX, 10 mM of PTX, and 15 μM of STT, or combinations, for 48 h. Data are represented as the mean ± the SD of three independent experiments, each carried out in triplicate. Statistical analysis: Mann–Whitney *U* test, * *p* < 0.05 vs. UCG; # *p* < 0.05 vs. DTX 50 nM and * *p* < 0.05 in the comparison between groups.

Given the fundamental role played by caspases in executing apoptosis, we decided to evaluate the effect of treatments on pan-caspase activation after 48 h of exposure. All treated groups presented higher activity of these enzymes with respect to the UCG (6.5% of positive cells). Consistent with the observations in the apoptosis assays, we found that the combination of PTX + STT exhibited significantly higher activation values of pan-caspases than the rest of the groups (Figure 5A). Additionally, to possess more elements in order to clarify which molecular pathway is initiating apoptosis under our experimental conditions, we evaluated the activation of the initiator caspases-8 and -9, representative of the extrinsic and mitochondrial pathways, respectively, as well as of the executor caspase-3. Statistical analysis revealed that, compared to the UCG, all treatments significantly positively affected the activation of each studied caspase (*p* < 0.05). Concerning caspase-3 and caspase-8 activities, the highest activation percentages were observed in PTX + STT + DTX and PTX + STT, with ranges near 80% (Figure 5B,C). In the case of caspase-9, we found that the best percentages of apoptosis were achieved in all STT-treated groups, principally in the STT + DTX and PTX + STT + DTX groups, with values above 95%, strongly suggesting that the mitochondrial pathway plays an important role in our observations (Figure 5D).

### 3.6. Increased Loss of Mitochondrial Membrane Potential (Δψm) after In Vitro Treatment of DU-145 Cells with Pentoxifylline, Stattic, Docetaxel, or Combinations

**Figure 6:**
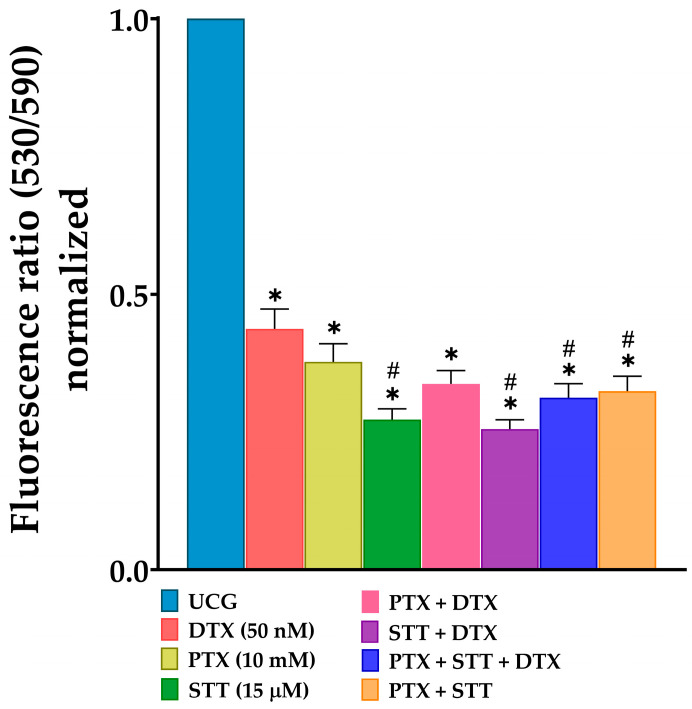
Effect of PTX, STT, DTX, or a combination on disrupting mitochondrial membrane potential (Δψm) in DU-145 cells. UCG = untreated control group; DTX = docetaxel; PTX = pentoxifylline; STT = Stattic. Cells were exposed to the treatments with 50 nM of DTX, 10 mM of PTX, and 15 μM of STT, or combinations, for 48 h, followed by measurement of Δψm using the JC-10 fluorescent reagent by spectrophotometry. Data are represented as the mean ± the SD of three independent experiments, each carried out in triplicate. Statistical analysis: Mann–Whitney *U* test, * *p* < 0.05 vs. UCG and # *p* < 0.05 vs. DTX 50 nM.

Alterations in the Δψm are one of the first steps that trigger the activation of apoptosis through the intrinsic pathway. For this reason, we decided to evaluate how treatments modified this. The results obtained after 48 h of treatment (Figure 6) showed that both individual treatments or combinations led to a significant loss of Δψm, with values close to 50%, compared to the UCG (*p* < 0.05), which exhibited an active mitochondrial membrane potential, a characteristic of cancer cells, with the STT and STT + DTX groups that caused a slightly more marked alteration. Taking these results together with those obtained with caspase-9 activity indicates that the apoptosis we observed is mainly triggered through the intrinsic pathway and suggests that STT is more active.

### 3.7. DU-145 Cells Were Arrested Predominantly in the G1 Phase of the Cell Cycle after Treatments with Pentoxifylline + Stattic + Docetaxel

**Figure 7:**
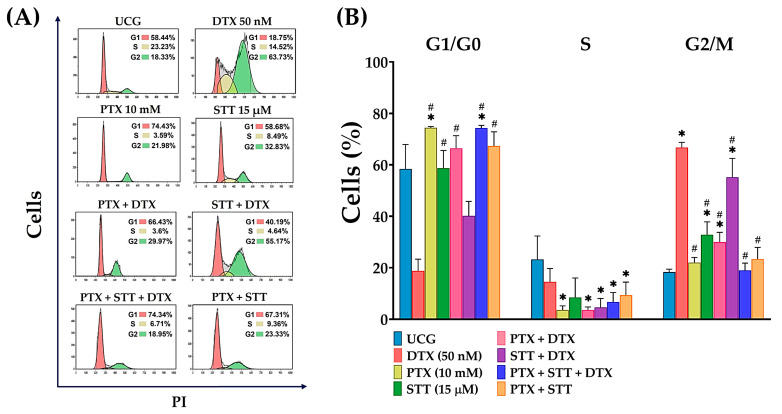
Effect of PTX, STT, DTX, or a combination on the cell cycle phases of DU-145 cells. UCG = untreated control group; DTX = docetaxel; PTX = pentoxifylline; STT = Stattic. (**A**) Representative histograms of DU-145 distribution in cell cycle phases from 1–3 independent experiments, and (**B**) quantification determined by flow cytometry after treatment with 50 nM of DTX, 10 mM of PTX, and 15 μM of STT, or combinations, for 48 h. Statistical analysis: Mann–Whitney *U* test, * *p* < 0.05 vs. UCG and # *p* < 0.05 vs. DTX 50 nM.

Cell cycle analysis was performed by flow cytometry using the change in DNA content through its stages. The majority of the cells of the different treatment groups accumulated during the G1 phase. However, the highest percentages were found in the PTX group (74.4% ± 0.5) and in the PTX + STT + DTX combination (74.3% ± 1.0), which were significantly different (*p* < 0.05) from those of the UCG group (58.4% ± 9.5). In the analysis of the S phase, we observed the lowest percentages of cells, and only significant differences (*p* < 0.05) were found when comparing the remaining treated groups with DTX (14.5% ± 5.2) and the UCG (23.2% ± 9.0). As for the G2 phase, the group treated with DTX revealed the highest percentages (66.7% ± 2.0) compared to all of the other groups (*p* < 0.05). Moreover, the combination of PTX + DTX modified the action of the chemotherapeutic drug in the cell cycle phase, arresting more cells in the G1 phase and giving rise to an effect similar to that shown in the PTX + STT + DTX group. On the other hand, for the STT + DTX combination, although the percentage of cells accumulated mainly during the G2 phase (55.18% ± 7.3), a critical percentage was also arrested in the G1 phase (40.2% ± 5.6) (Figure 7).

### 3.8. Increased Activity of Proapoptotic Genes and Decreased Expression of Antiapoptotic Genes in DU-145 Cells after Exposure to Pentoxifylline + Stattic in the Presence or Not of Docetaxel

**Figure 8:**
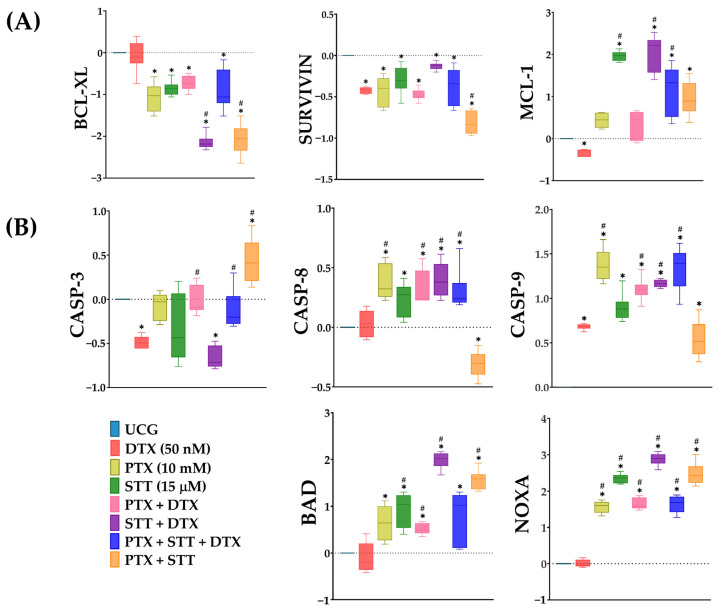
Effect of PTX, STT, and DTX, or a combination, on anti- and proapoptotic gene expression in DU-145 cells. UCG = untreated control group; DTX = docetaxel; PTX = pentoxifylline; STT = Stattic. Cells were exposed to the treatments with 50 nM of DTX, 10 mM of PTX, and 15 μM of STT, or combinations, for 4 h. Graphs depict the relative expression (Log2FC) levels of (**A**) antiapoptotic genes: *BCL-XL*, *SURVIVIN*, and *MCL-1*, and (**B**) proapoptotic genes: *BAD*, *NOXA*, *CASP-3*, *CASP-8*, and *CASP-9*, measured using the LightCycler version 2.0 PCR system. The UCG group was used as a calibrator, and *RPS18* and *RPLP0* were utilized as reference genes. Data are represented as the mean ± the SD. Statistical analysis: Mann–Whitney *U* test, * *p* < 0.05 vs. UCG and # *p* < 0.05 vs. DTX 50 nM.

We also aimed to assess alterations in the expression of some apoptosis-related genes, which can either inhibit (such as *BCL-XL*, *SURVIVIN*, and *MCL-1*) or promote (including *BAD*, *NOXA*, *CASP-3*, *CASP-8*, and *CASP-9*) apoptosis in response to the treatments. Our findings indicated significant modifications in the relative expression of pro- and antiapoptotic genes (*p* < 0.05) (Figure 8). With respect to proapoptotic genes, all demonstrated elevated expression levels except for *CASP-3*. Notably, *BAD*, *NOXA*, and *CASP-9* exhibited the most significant overexpression in response to the treatments, principally in the STT-treated groups. Regarding the antiapoptotic genes *BCL-XL* and *SURVIVIN*, we observed a significant downregulation in both genes, unlike *MCL-1*, which exhibited resistance to the treatments, demonstrating upregulation in all groups except in the DTX group. Overall, it was observed that the combined treatments of PTX + STT + DTX and PTX + STT exerted a higher effect in upregulating proapoptotic genes and inhibiting antiapoptotic genes.

### 3.9. Pentoxifylline and Stattic Treatments Alone and Mostly Combined Led to a Significant Decrease in p65 and STAT-3 Phosphorylation in DU-145 Cells

**Figure 9:**
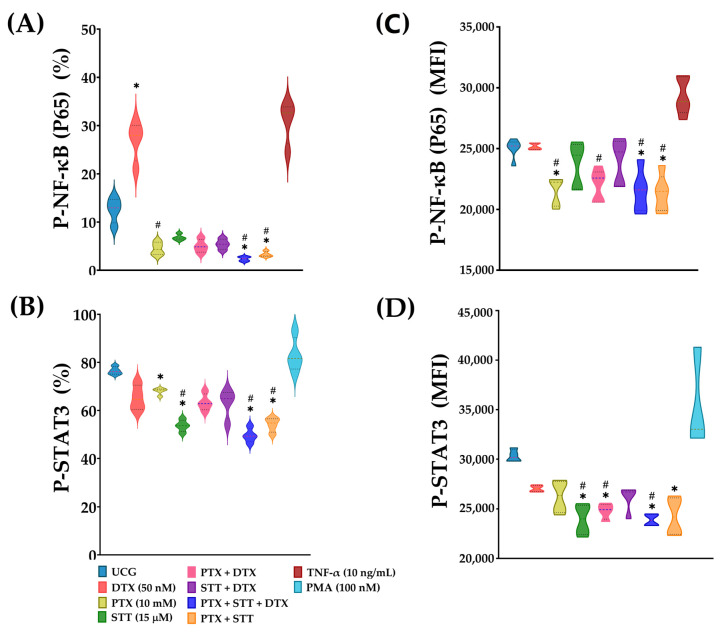
Effect of PTX, STT, DTX, or a combination on the phosphorylation of NF-κB-p65 (Ser536) and STAT-3 (Tyr705) in DU-145. UCG = untreated control group; DTX = docetaxel; PTX = pentoxifylline; STT = Stattic. Cells were treated with 50 nM DTX, 10 mM PTX, and 15 μM STT, as well as a combination, for 1 h. To provide the experiment with greater reliability, the cells were treated with 10 ng/mL tumor necrosis factor-alpha (TNF-α) and with 100 nM phorbol myristate acetate (PMA) for 30 min as positive controls of p65 and STAT-3 phosphorylation, respectively. Results are displayed as the percentage of cells positive for p65 (**A**) and STAT3 (**B**) phosphorylation, as well as their respective mean fluorescence intensity (MFI) values (**C**,**D**), representing the mean ± the SD. Statistical analysis: Mann–Whitney *U* test, * *p* < 0.05 vs. UCG and # *p* < 0.05 vs. DTX 50 nM.

Our work is based on the inhibition of NF-κB and STAT-3. For this reason, we determined the phosphorylation of p65 from NF-κB, and Tyr705 phosphorylation from STAT-3, under our experimental conditions. Figure 9 illustrates, as expected, that DTX strongly incremented the phosphorylation of p65 as a response to chemotherapeutic injury. In the experimental group (PTX + STT + DTX), a trend of declining phosphorylation of both molecules was induced by PTX and STT, respectively, with a significant reduction noticeable in both the percentage of phosphorylated cells as well as in a lesser density of molecules, as indicated by the MFI graphics (Figure 9C,D). The results were similar to those in the other treatment groups. Taken together, these results support the idea that the observations of the present work are due, at least in part, to the inactivation of the NF-κB and STAT-3 transcription factors.

## 4. Discussion

The concept of chemotherapy with a rational molecular basis involves targeting specific points on tumor cells to increase their sensitivity to chemotherapy or to induce apoptosis directly, and the use of two or more molecules with different action mechanisms is a common practice in oncology. Here, we combine traditional chemotherapy with molecular targets; commonly, a sole molecular target is employed in these studies. In this study, we attacked two molecular targets. This study validates this approach and underscores its significance by confirming previous findings. By inhibiting two key transcription factors, NF-κB and STAT-3, with PTX and STT, we demonstrate that dual blockade is more effective than targeting either factor separately.

The findings in the present study align with prior research and demonstrate agreement between them with clear statistical differences that provide assurance to the work. We selected the DU-145 cells based on previous evidence indicating significant hyperactivation of the NF-κB and STAT-3 signaling pathways [31,32,33]. Furthermore, this permitted us to investigate drug efficacy in vitro under more rigorous conditions, given that the DU-145 line corresponds to a metastatic castration-resistant human PCa model, which in turn represents a remarkably complex therapeutic challenge [3,34]. Our observations are reliable because the difference among the groups is clear, there is good correlation among the different experiments carried out in the present, and the observations agree with the results found in the literature.

Chemotherapy is widely recognized for its ability to induce apoptosis in tumor cells, leading to their destruction [35]. Therefore, we initially determined a dose–response curve based on previous work to determine the ideal doses for the assays [30,36]. We observed that the drugs revealed a dose–response effect, suggesting the specificity of the mechanism of action (Figure 1). It is important to stress that the doses used for both inhibitors fall within the range of the published doses in different tumor cell lines [37,38,39,40]. We observed that DTX had a lower effect on inducing DU-145 cells into apoptosis compared to the other treated groups, especially those treated with STT. However, when inhibitor drugs were added to the DTX therapy, apoptosis significantly increased, with the triple combination PTX + STT + DTX achieving the best rates, with clear statistical significance (*p* < 0.05), at both time points studied, confirming the hypothesis that the best therapy is the blockage of two molecular targets at the same time (Figure 2). Notably, a comparable effect was obtained despite excluding DTX and treating only with PTX + STT. Similar results have been published in different tumor cells treated with either PTX or STT [30,41]. This is important because it opens the possibility of different treatments with fewer side effects and ones that are less expensive.

Another important point is that these combinations achieved a greater induction of apoptosis during the late stage compared to the other treatments. This is noteworthy because it represents a point of no return for this mechanism of cell death [42,43]. One additional aspect to highlight is that these combinations of PTX + STT with or without DTX achieve the most effective antiproliferative effects in DU-145 cells (Figure 3), and that all of these results taken together confirm the hypothesis that two inhibitors are better than one.

In addition, the observations on apoptosis in our study are related to the activation of caspases, and we found that groups exhibiting a more significant apoptotic effect were those with a higher activity of these enzymes. Furthermore, the graphs depict that the intrinsic and extrinsic pathways are involved at the same level (Figure 5C,D). Nevertheless, the dramatic loss of mitochondrial membrane potential obtained with these groups (Figure 6) suggests a greater involvement of caspase-9.

With respect to the cell cycle results (Figure 7), it is noteworthy that the cells that received PTX + STT and the triple-combination treatment demonstrated a marked arrest during the G1 phase, because it has been reported that at this point, the cells become more sensitive to the toxicity of antitumor therapies [44]. Moreover, fewer cells during the G2 phase have a lesser opportunity to use the mechanisms to repair their DNA [45,46]; therefore, our results suggest that a “double strike” would be delivered against the cells that favor the action of the treatments. Conversely, the DTX-treated group revealed the highest percentages of cells arrested in G2 compared to all of the remaining groups, which was to be expected, in that it is a cell cycle-specific chemotherapeutic agent that inhibits mitotic spindle formation [47].

We were also interested in discovering what was happening at the transcriptional level once the DU-145 PCa cells were treated. The present results show that the balance between the anti- and proapoptotic genes favors apoptosis (see Figure 8), because we observed a marked decrease in the expression of the antiapoptotic genes *BCL-XL* and *SURVIVIN*. At the same time, the levels of *BAD*, *NOXA*, *CASP-3*, -*8*, and *-9* increased mainly under PTX + STT + DTX and PTX + STT treatments, with an exception encountered in the latter group in terms of *CASP-8* expression.

Another striking observation was found in the clonogenic assay. This experiment was performed 4 h after treatment because the high mortality presented during more extended periods prevented it from being conducted. Figure 4 shows that all treatments affect the clonogenic capacity of tumor cells. The fact that the groups receiving PTX + STT exhibited a higher inhibition, one near 100%, which is the same as that found in all groups containing STT, suggests that this inhibitor is responsible for clonogenic inhibition; this is supported by the fact that PTX alone was not as effective as what was observed in these groups. This significant effectiveness shown by STT in decreasing the clonogenic capacity of DU-145 PCa cells has already been reported [33].

Last, when we analyzed the results obtained in determining the phosphorylation status of NF-κB and STAT-3, we could ascertain that PTX and STT treatments effectively led to the downregulation of these transcription factors (Figure 9). As we expected, the synthetic inhibitor of STAT-3, STT, led to a significant decline in the expression of this factor. Interestingly, to a lesser extent, we could also identify repressor activity of STAT-3 by PTX, something previously reported by Kamran et al. in melanoma cells [48]. This might represent an additional mechanism that could explain the effect we found with the combination of both drugs, but this must be confirmed. However, downregulation of this factor continued to be observed when DTX was combined with PTX or STT, which might represent a reinforcement for maintaining the apoptotic mechanisms activated, thus eradicating DU-145 PCa cells.

Considering the above, it is possible to explain the observations obtained with this study. Direct inhibition of NF-κB and STAT-3 by PTX and STT leads to a balance that favors apoptosis [11]; therefore, DU-145 PCa cells entertain less opportunity to defend themselves. The aforementioned indicates that the joint use of these drugs potentiates the effect of each of them (Supplementary Figure S1 and Table S1), resulting in a more significant increase in apoptosis compared to treatments administered individually.

However, although this study constitutes a first exploration that analyzes the potential of the simultaneous blockade of NF-κB and STAT-3 with these drugs on the induction of apoptosis in a metastatic PCa cell line with persistent activity of these pathways and provides evidence of this, its limitations need to be acknowledged. In particular, using a single cell line may limit the generalizability of the findings. Furthermore, it is essential to carry out future in vivo studies with appropriate protocols to examine the effect of these drugs in a more complex physiological setting, allowing a more robust validation and extension of these findings.

## 5. Conclusions

In this report, we show that it is possible to utilize two molecular inhibitors with promising results, even if they may be more effective than chemotherapy. In addition, this supports the concept of chemotherapy with rational molecular bases and opens the possibility of assaying a combination of different molecular inhibitors ad hoc for different tumors. Nevertheless, further in vivo studies are necessary to confirm these findings and assess their therapeutic potential.

## Figures and Tables

**Table 1:** Chemical structures of docetaxel, pentoxifylline, and Stattic.

PubChem ID	Name and Abbreviation	Structure
CID 148124	Docetaxel DTX	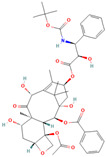
CID 4740	Pentoxifylline PTX	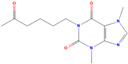
CID 2779853	Stattic STT	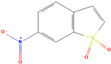

CID = PubChem’s compound identifier, a non-zero integer for a unique chemical structure.

**Table 2:** Primer sequences.

Gene	Primer Sequence (5′→3′)	Amplicon Length	GenBank Accession No.
*BCL-XL*	F: GCA GGC GAC GAG TTT GAA CT R: GTG TCT GGT CAT TTC CGA CTG A	434 bp	NM_001191
*SURVIVIN*	F: TGA GCT GCA GGT TCC TTA TCT G R: GAA TGG CTT TGT GCT TAG TTT T	234 bp	NM_001168
*MCL-1*	F: CAC GAG ACG GTC TTC CAA GGA TGC T R: CTA GGT TGC TAG GGT GCA ACT CTA GGA	497 bp	NM_021960
*BAD*	F: CTC CGG AGG ATG AGT GAC GAG T R: ACT TCC GCC CAT ATT CAA GAT	240 bp	NM_004322
*NOXA*	F: GAG ATG CCT GGG AAG AAG G R: TCC TGA GCA GAA GAG TTT GGA	162 bp	NM_021127
*CASP-3*	F: ATA CTC CAC AGC ACC TGG TTA T R: AAT GAG AGG ATA CAG TAC CAA	329 bp	NM_004346
*CASP-8*	F: ACC TGC TGG ATA TTT TCA TAG AGA R: TGT TGA TGA TCA GAC AGT ATC CC	264 bp	NM_001228
*CASP-9*	F: GTA CGT TGA GAC CCT GGA CGA C R: GTC GCT AAG AGC CTG TCT GTC ACT	323 bp	NM_001229
*RPS18*	F: CGA TGG GCG GCG GAA AA R: CAG TCG CTC CAG GTC TTC ACG G	117 bp	NM_022551
*RPL32*	F: GCA TTG ACA ACA GGG TTC GTA G R: ATT TAA ACA GAA AAC GTG CAC A	320 bp	NM_000994

F = forward; R = reverse; bp = base pairs.

## Data Availability

The datasets used and/or analyzed during the current study are available from the corresponding author upon reasonable request.

## Supplementary Materials

The following supporting information can be downloaded at: https://www.mdpi.com/article/10.3390/cimb46090605/s1. Figure S1: effects of Pentoxifylline (PTX) combined with Stattic (STT) were evaluated in DU-145 cells after 24 h treatment using Synergy Finder version 3 software; (**A**) Dose-response matrix of the percentage of cell growth inhibition; (**B**) Highest Single Agent (HSA) diagram to identify the areas that point to synergistic combinations between PTX and STT; (**C**) Calculation and visualization of synergy scores for the PTX and STT combinations. Data are from three independent experiments performed in triplicate. Synergy Score: Less than −10: the interaction between two drugs is likely to be *antagonistic*; From −10 to 10: the interaction between two drugs is likely to be *additive*; Larger than 10: the interaction between two drugs is likely to be *synergistic.* Table S1: Synergy scores for PTX combined with STT in treatment on DU-145 cells after 24 h using Synergy Finder version 2 software. Data are from three independent experiments performed in triplicate. Synergy Score: Less than −10: the interaction between two drugs is likely to be *antagonistic*; From −10 to 10: the interaction between two drugs is likely to be *additive*; Larger than 10: the interaction between two drugs is likely to be *synergistic.*
