# Supplementary material for: Improvement of Docetaxel Efficacy through Simultaneous Blockade of Transcription Factors NF-κB and STAT-3 Using Pentoxifylline and Stattic in Prostate Cancer Cells
Source: Curr Issues Mol Biol. 2024 Sep 14;46(9):10140–59. doi: 10.3390/cimb46090605 (PMC11431379; doi:10.3390/cimb46090605)
Supplement: Supplementary file 1 [file cimb-46-00605-s001.zip › cimb-3172609-supplementary.pdf]

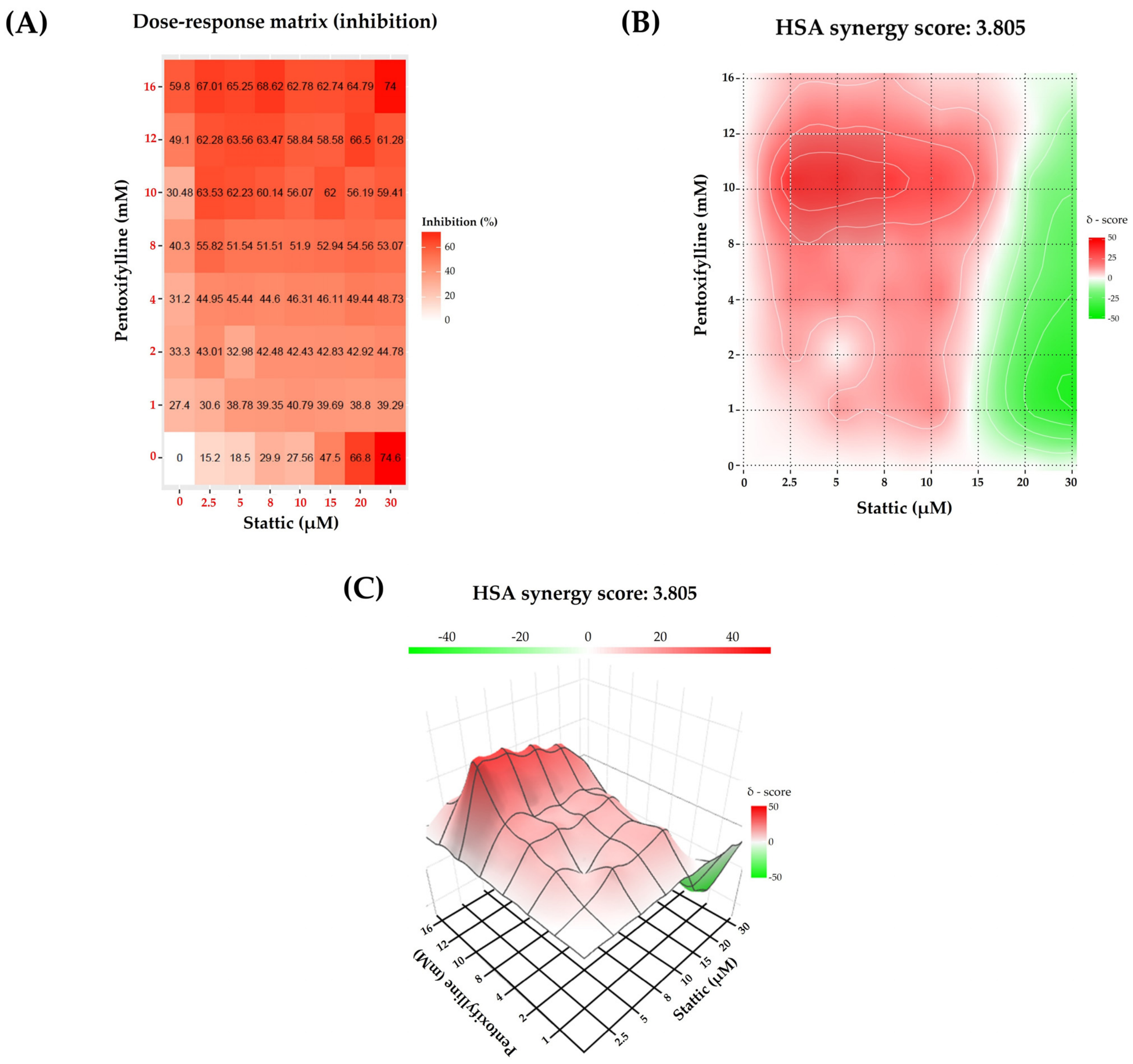

Drug combination	Synergy score	Most synergistic area score	Method
Stattic - Pentoxifylline	3.81	23.55	HSA

Supplementary Figure S1. Synergistic effects of Pentoxifylline (PTX) combined with Stattic (STT) were evaluated in DU-145 cells after 24 h treatment using Synergy Finder version 3 software; (**A**) Dose-response matrix of the percentage of cell growth inhibition; (**B**) Highest Single Agent (HSA) diagram to identify the areas that point to synergistic combinations between PTX and STT; (**C**) Calculation and visualization of synergy scores for the PTX and STT combinations. Data are from three independent experiments performed in triplicate. Synergy Score: Less than -10: the interaction between two drugs is likely to be *antagonistic*; From -10 to 10: the interaction between two drugs is likely to be *additive*; Larger than 10: the interaction between two drugs is likely to be *synergistic*.

Supplementary Table S1. Synergy scores for PTX combined with STT in treatment on DU-145 cells after 24 h using Synergy Finder version 2 software. Data are from three independent experiments performed in triplicate. Synergy Score: Less than -10: the interaction between two drugs is likely to be *antagonistic*; From -10 to 10: the interaction between two drugs is likely to be *additive*; Larger than 10: the interaction between two drugs is likely to be *synergistic*.

Pair Index	Drug 1	Drug 2	Conc1 (mM)	Conc 2 (µM)	Relative inhibition	Synergy
1	Pentoxifylline	Stattic	0	0	0	0
1	Pentoxifylline	Stattic	1	0	27.4	0
1	Pentoxifylline	Stattic	2	0	33.3	0
1	Pentoxifylline	Stattic	4	0	31.2	0
1	Pentoxifylline	Stattic	8	0	40.3	0
1	Pentoxifylline	Stattic	10	0	30.48	0
1	Pentoxifylline	Stattic	12	0	49.1	0
1	Pentoxifylline	Stattic	16	0	59.8	0
1	Pentoxifylline	Stattic	0	2.5	15.2	0
1	Pentoxifylline	Stattic	1	2.5	30.6	3.887441482
1	Pentoxifylline	Stattic	2	2.5	43.01	11.79595525
1	Pentoxifylline	Stattic	4	2.5	44.95	16.70385012
1	Pentoxifylline	Stattic	8	2.5	55.82	18.85409119
1	Pentoxifylline	Stattic	10	2.5	63.53	40.14998156
1	Pentoxifylline	Stattic	12	2.5	62.28	16.0113996
1	Pentoxifylline	Stattic	16	2.5	67.01	8.758891589
1	Pentoxifylline	Stattic	0	5	18.5	0
1	Pentoxifylline	Stattic	1	5	38.78	13.82471377
1	Pentoxifylline	Stattic	2	5	32.98	-0.388744148
1	Pentoxifylline	Stattic	4	5	45.44	17.2991146
1	Pentoxifylline	Stattic	8	5	51.54	13.65463821
1	Pentoxifylline	Stattic	10	5	62.23	38.57070846
1	Pentoxifylline	Stattic	12	5	63.56	17.5663762
1	Pentoxifylline	Stattic	16	5	65.25	6.620798774
1	Pentoxifylline	Stattic	0	8	29.9	0
1	Pentoxifylline	Stattic	1	8	39.35	11.48010063
1	Pentoxifylline	Stattic	2	8	42.48	11.15209775
1	Pentoxifylline	Stattic	4	8	44.6	16.27866121
1	Pentoxifylline	Stattic	8	8	51.51	13.61819344
1	Pentoxifylline	Stattic	10	8	60.14	36.03172324
1	Pentoxifylline	Stattic	12	8	63.47	17.45704191
1	Pentoxifylline	Stattic	16	8	68.62	10.71476059
1	Pentoxifylline	Stattic	0	10	27.56	0
1	Pentoxifylline	Stattic	1	10	40.79	16.07214088
1	Pentoxifylline	Stattic	2	10	42.43	11.09135648
1	Pentoxifylline	Stattic	4	10	46.31	18.35601275
1	Pentoxifylline	Stattic	8	10	51.9	14.09197537

1	Pentoxifylline	Stattic	10	10	56.07	31.0873836
1	Pentoxifylline	Stattic	12	10	58.84	11.83240001
1	Pentoxifylline	Stattic	16	10	62.78	3.62017988
1	Pentoxifylline	Stattic	0	15	47.5	0
1	Pentoxifylline	Stattic	1	15	39.69	-9.487786867
1	Pentoxifylline	Stattic	2	15	42.83	-5.673234913
1	Pentoxifylline	Stattic	4	15	46.11	-1.688607394
1	Pentoxifylline	Stattic	8	15	52.94	6.60865052
1	Pentoxifylline	Stattic	10	15	62	**17.61496922**
1	Pentoxifylline	Stattic	12	15	58.58	11.51654539
1	Pentoxifylline	Stattic	16	15	62.74	3.571586862
1	Pentoxifylline	Stattic	0	20	66.8	0
1	Pentoxifylline	Stattic	1	20	38.8	-34.01511297
1	Pentoxifylline	Stattic	2	20	42.92	-29.01003206
1	Pentoxifylline	Stattic	4	20	49.44	-21.08937004
1	Pentoxifylline	Stattic	8	20	54.56	-14.86946367
1	Pentoxifylline	Stattic	10	20	56.19	-12.88929816
1	Pentoxifylline	Stattic	12	20	66.5	-0.364447639
1	Pentoxifylline	Stattic	16	20	64.79	-2.441799181
1	Pentoxifylline	Stattic	0	30	74.6	0
1	Pentoxifylline	Stattic	1	30	39.29	-42.8954871
1	Pentoxifylline	Stattic	2	30	44.78	-36.22609531
1	Pentoxifylline	Stattic	4	30	48.73	-31.42753473
1	Pentoxifylline	Stattic	8	30	53.07	-26.15519222
1	Pentoxifylline	Stattic	10	30	59.41	-18.45319879
1	Pentoxifylline	Stattic	12	30	61.28	-16.18147517
1	Pentoxifylline	Stattic	16	30	74	-0.728895278

* Conc = Concentration of drug used.
